# Preliminary Study to Understand the Role of Gut Microbiota in Coronary Slow Flow Phenomenon (CSFP)

**DOI:** 10.3390/metabo15070475

**Published:** 2025-07-14

**Authors:** Tayfun Gurol, Tayyip Karaman, Yesim Gurol, Osman Ugur Sezerman, Sinem Oktem Okullu

**Affiliations:** 1 Department of Cardiology, Faculty of Medicine Maltepe University, Istanbul 34857, Turkey; tayfun.gurol@maltepe.edu.tr; 2 Department of Medical Biotechnology, Acibadem Mehmet Ali Aydinlar University, Istanbul 34752, Turkey; tayyipkaramannn@gmail.com (T.K.); dryesimg@gmail.com (Y.G.); 3 Department of Biostatistics and Medical Informatics, School of Medicine, Acibadem Mehmet Ali Aydinlar University, Istanbul 34752, Turkey; ugur.sezerman@acibadem.edu.tr; 4 Department of Medical Microbiology, School of Medicine, Acibadem Mehmet Ali Aydinlar University, Atasehir, Istanbul 34752, Turkey

**Keywords:** coronary slow flow phenomenon, coronary health, microbiome

## Abstract

**Background/Objectives:** Coronary slow flow phenomenon (CSFP) is a cardiovascular condition characterized by delayed passage of contrast medium through the coronary arteries, predominantly affecting young male smokers admitted with acute coronary syndrome. Although over 80% of patients experience recurrent chest pain and more than 20% require readmission, the etiology of CSFP remains poorly understood. Given the emerging role of gut microbiome in cardiovascular diseases, this study investigates the microbial composition associated with CSFP. **Methods:** Stool samples were collected from patients diagnosed with CSFP and healthy control individuals. Microbiota profiling was performed using 16S rRNA sequencing. Taxonomic differences were evaluated to identify microbial markers potentially associated with CSFP. **Results:** The analysis revealed a notable enrichment of the genus *Gemmiger* and the species *Anaerobutyricum* in CSFP patients, specifically within the selenium metabolism pathway. This is of particular interest given the established link between selenium deficiency and heightened cardiovascular risk, suggesting a possible microbiome-mediated modulation of selenium bioavailability in CSFP pathophysiology. Moreover, a marked increase in taxa associated with the biosynthesis of trimethylamine (TMA), a proatherogenic metabolite implicated in the onset and progression of various cardiovascular disorders, was observed in the CSFP cohort, further supporting a potential mechanistic role of gut microbiota in the disease’s underlying etiology. **Conclusions:** Although statistical significance could not be established due to the limited sample size, the observed trends support the hypothesis that specific gut microbes and metabolic pathways, particularly those linked to selenium metabolism and TMA production, may serve as potential microbial indicators for CSFP. These preliminary findings warrant further investigation in larger cohorts.

## 1. Introduction

Coronary angiography is the gold standard diagnostic method used in the diagnosis of coronary artery disease. Although there is no severe stenosis in the epicardial coronary arteries in 1% to 5% of coronary angiographies, the flow rate of the opaque material in the coronary arteries decreases [1]. This phenomenon is called the coronary slow flow phenomenon. Coronary slow flow phenomenon is one of the causes of cardiac chest pain. This may be the reason for hospitalization due to myocardial ischemia, myocardial infarction, arrhythmias, and recurrent chest pain [2]. Although there are various theories and hypotheses about the pathophysiology of coronary slow flow, endothelial dysfunction is the strongest among them. Owing to endothelial dysfunction, the balance between the release of vasodilating nitric oxide and the release of vasoconstricting endothelin is disrupted, and the underlying cause of coronary slow flow is thought to occur [3]. Causes of endothelial dysfunction include classical causes such as advanced age, hypertension, diabetes mellitus, hyperlipidemia, smoking, genetic factors, obesity, increased CRP, and some microorganisms that cause inflammation [4]. Many viruses, including SARS-CoV-2, have been reported to cause endothelial dysfunction [5].

Next-generation sequencing provides a better understanding of the human gut microbiome and its association with diseases such as cardiovascular diseases, obesity, diabetes, cancer, and neurological disorders [6]. The gut microbiome of patients with cardiovascular diseases and healthy control individuals has been widely studied. An increasing amount of *Faecalibacterium prausnitzii* and a significant decrease in the population of *Ruminococcus gravus* were found to be correlated with heart failure [7]. Moreover, an increase in *Prevotella* and *Klebsiella* genera is a potential biomarker of hypertension [8]. In addition, *Escherichia coli*, *Klebsiella* spp., *Enterobacter aerogenes*, *Streptococcus* spp., *Lactobacillus salivarius*, *Solobacterium moorei*, *Atopobium parvulum*, *Ruminococcus gnavus*, and *Eggerthella lenta* are positively correlated with atherosclerotic cardiovascular diseases. In contrast, *Roseburia intestinalis*, *Faecalibacterium* cf. *prausnitzii*, *Bacteroides* spp., *Prevotella copri*, and *Alistipes shahii* are negatively correlated with atherosclerosis [9]. All these associations indicate the importance of microbiome studies; however, without knowledge of microbiome pathways and their contributions to disease progression, these correlations will remain deficient.

CSFP is not a rare finding in patients undergoing routine coronary angiography. Coronary slow flow has been reported in 1% of patients who underwent coronary angiography with a diagnosis of acute coronary syndrome [10]. On the other hand, in another study, slow coronary flow was observed in 7% of patients who presented with chest pain and underwent angiography with the suspicion of coronary artery disease [11]. It has been associated with significant morbidity and possibly mortality. Further studies are necessary for effective treatment [12]. CSFP was first observed during angiographic imaging [13]. The thrombolysis flow rate chart in myocardial infarction is used to indicate coronary blood flow. It shows the speed of the passage of the injected contrast agent in the coronary artery and whether it is complete [14]. It is characterized by slow passage of contrast medium without stenosis in some patients with chest pain who are receiving selective coronary angiography. This phenomenon has been named the slow flow phenomenon, which was defined by Tambe in 1972 [11,15]. It is undeniable that coronary slow flow is still referred to as a phenomenon that is not an innocent entity. Unless there are prospective objective methods and studies investigating the effect of coronary slow flow on ventricular function, CSFP will remain a phenomenon for a long time, perhaps the opposite of what it deserves.

The gut microbiome plays a pivotal role in modulating host physiology and has been increasingly recognized for its influence on the onset and progression of various diseases, including those affecting the cardiovascular system. Despite this, the relationship between gut microbial composition and coronary slow flow phenomenon (CSFP) remains unexplored. CSFP, often considered a precursor to more severe cardiovascular conditions such as atherosclerosis, is characterized by delayed coronary blood flow in the absence of significant arterial obstruction, and its underlying mechanisms are not yet fully elucidated. Given the growing evidence linking gut microbial dysbiosis to cardiovascular pathology, this study aimed to investigate whether distinct differences exist in the gut microbiota profiles of CSFP patients compared to healthy individuals, thereby uncovering potential microbial contributions to the pathophysiology of CSFP.

## 2. Materials and Methods

### 2.1. Sample Collection and Patient Cohort

This preliminary study was conducted on 8 adults at the age of 50 (SD ± 7), comprising four CSFP patients and four healthy controls for investigating the differences between gut microbial communities of two groups. Pair matching was considered while constructing the groups to limit the possibility of independent variables such as age, diet, and lifestyle. Those who had distinct lifestyles and bad habits (smoking, alcohol consumption, diet, exercise, etc.), those that recently used antibiotics, antidepressants, and other drugs that directly affect the microbial community changes in the gut were excluded from the study. The control group’s inclusion criteria included no association with cardiovascular diseases and an age of older than 40.

Fecal samples were collected from each participant into storage tubes that contain DNA/RNA Shield solution to protect the microbial community in fecal samples. DNA isolation was performed immediately after it arrived to laboratory. The gDNA extraction process was carried out with a ZymoBIOMICS DNA Mini-prep kit (Zymo Research Corp., Irvine, CA, USA; Cat. No. D4300). The concentrations and purity levels were measured with an HS dsDNA Qubit 2.0 (Thermo Fisher Scientific, Waltham, MA, USA; Cat. No. Q32866) and a NanoDrop OneC (Thermo Fisher Scientific, Waltham, MA, USA; Cat. No. ND-ONE-W), respectively. Isolated DNA samples were kept at −20 °C for sequencing.

### 2.2. 16S rRNA Microbiome Sequencing Analysis

The extracted DNA samples were subjected to 16S rRNA microbiome and pathway prediction analyses. The Oxford Nanopore Technology (ONT) sequencing platform was used to perform the sequencing assay and analyze the gut microbial community of coronary slow flow syndrome patients via 16S targeted sequencing, which covers all variable regions of the 16S rRNA gene (V1–V9). The gDNA obtained from DNA extraction was subjected to PCR for targeted 16S rRNA gene region sequencing via the ONT 16S barcoding kit (Oxford Nanopore Technologies, Oxford, UK; Cat. No. SQK-16S024). Library preparation was performed according to the manufacturer’s instructions. Base calling conversion from fast5 signal files to fastq sequence data and adapter trimming were performed with the Guppy cli toolkit (v. 6.0.6). Quality control of each sample was carried out with FastQC (v. 0.11.2). Trimming and error correction were carried out with the bbtools cli toolkit (v. 38.97). The corrected reads were used to create consensus sequences via ncbi-magicblast (v 1.6.0). Consequently, prokaryotic annotation was carried out with blastn (v 2.13.0) to identify the microorganisms according to the NCBI 16S database (as of 27 March 2023).

### 2.3. Statistical Analysis

The R programming language was used for statistical analysis [16]. The OTU tables obtained from the preprocessing steps were standardized in R Studio (version 4.4.1) via the total sum scaling (TSS) approach. The normality of OTU count data was evaluated using Shapiro–Wilk test. The Mann–Whitney U test was performed for normalized out tables to determine the significant taxonomy differences between patients with CSFP and healthy subjects. Results were accepted as statistically significant if *p* value is less than 0.05. Significantly differentiated OTUs between control and healthy groups were visualized by violin plots generated by ggplot2 package (version 3.4.4.).

## 3. Results

The 16S rRNA gene is approximately 1.5 kb long, with 9 hypervariable regions from V1 to V9. The targeted sequencing assay used all the V1 to V9 rRNA gene regions. A total of 135016 reads were generated for 8 samples, and the N50 of the reads was calculated to be 1423 bp. The average quality of the samples was Q18, and the GC content of eight samples was 53%. 16S rRNA results were evaluated at phylum, genus, and species level.

**Figure 1:**
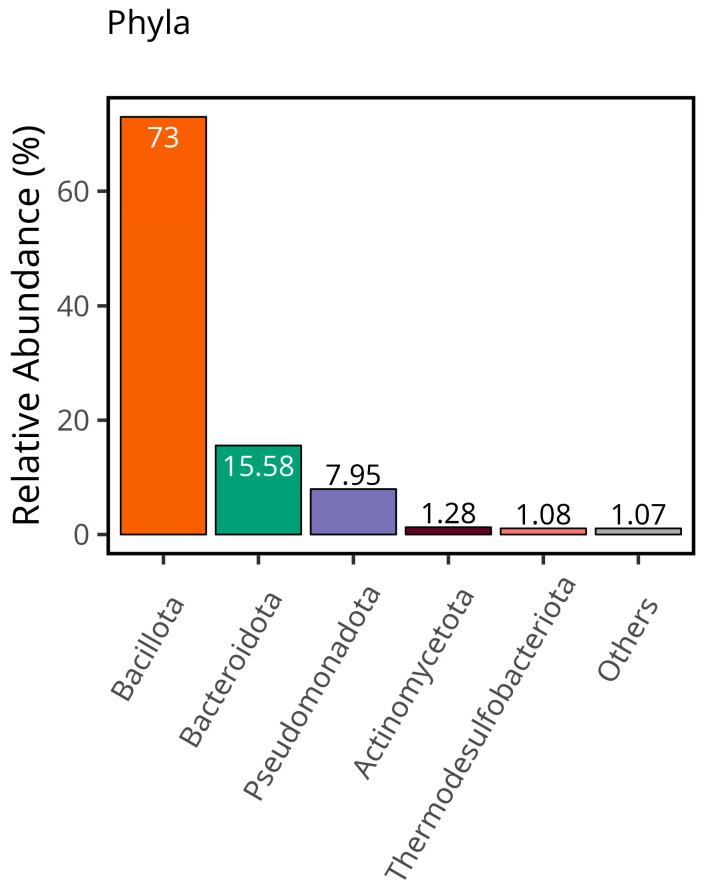
The total relative abundance of each phylum provides a comprehensive overview of the overall distribution within the dataset.

Overall distribution of samples indicated that the most abundant phyla was Bacillota with 73%, followed by *Bacteroidota* (15.58%), *Pseudomonadota* (7.95%), *Actinomycetota* (1.28%), *Thermodesulfobacteriota* (1.08%), and others (1.07%), as illustrated in Figure 1. Despite the result not being significant (*p* > 0.05), 5 OTUs were discovered to be explanatory for the CSFP patients: *Bacillota*, *Calditrichota*, *Lentisphaerota*, *Pseudomonadota*, and *Synergistota* (OTU4, OTU9, OTU26, OTU32, and OTU35 at the phylum level, respectively). Almost 90% of microbial consumption was occupied by *Bacillota* and *Bacteroidota* in disease and healthy groups (74% for disease group, 71% for control group, *p* > 0.05).

**Figure 2:**
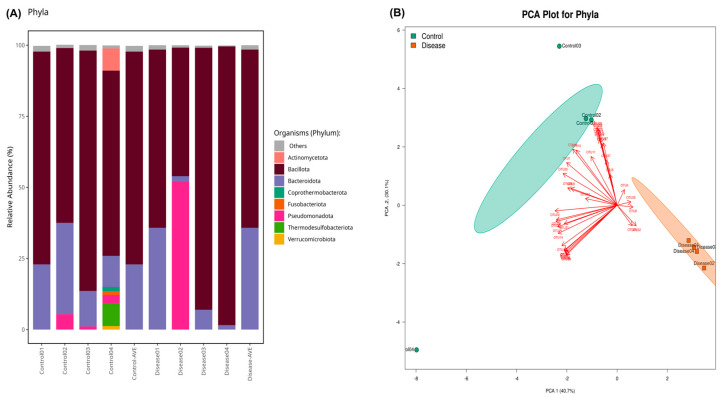
(**A**) The stacked bar plot indicates the relative abundance of phyla in the control and patient groups. “Control-AVE” refers to the average of the whole control group phylum, and “Disease-AVE” indicates the average of the whole disease group phylum. (Those that have a relative abundance value less than 1% were classified as others). (**B**) PCA results present the clustered healthy individuals (Control) and CSFP patient groups (Disease) as well as OTUs that provide the discrimination of both groups in a visual format. The green colors show healthy samples, and the red ones demonstrate the patient.

*Bacillota* was the most abundant phylum, followed by *Bacteroidota*, *Pseudomonadota* (13.3% for disease group, 2.6% for control group, *p* > 0.05), *Actinomycedota* (0.22% for disease group, 2.3% for control group, *p* > 0.05), *Thermodesulfobacteriota* (0.05% for disease group, 2.1% for control group, *p* > 0.05), and others, which was demonstrated in Figure 2A. Eight phyla (*Acidobacteriota, Balneolota, Campylobacterota, Cyanobacteriota, Elusimicrobiota, Myxococcota, Spirochaetota,* and *Thermotogota*) were identified as significantly more abundant in the control group compared to patients diagnosed with CSFP (*p* < 0.05). Principal component analysis was applied to the microbiome dataset, and the clustering of the control and patient groups was evaluated at the phylum level shown in Figure 2B. Discriminative OTUs that are only found in control groups provide a certain aggregate.

**Figure 3:**
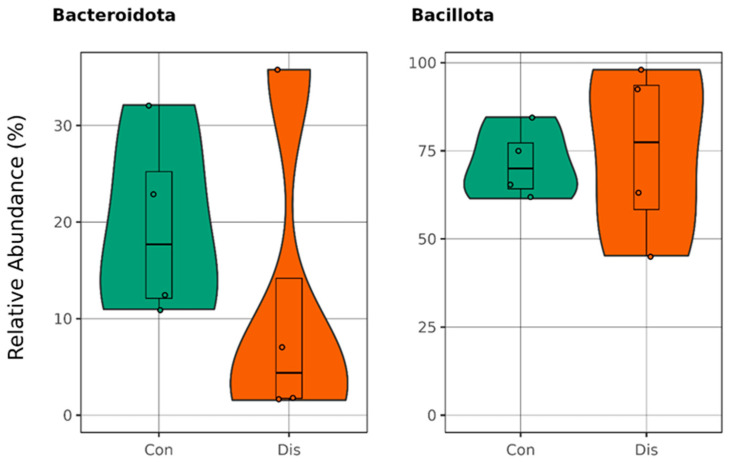
Boxplots illustrate the relative abundances in the phyla Bacillota and Bacteroidota, with the green plots representing healthy individuals in the control group (Con) and the orange plots depicting patients diagnosed with CSFP disease (Dis) (*p* > 0.05).

Conversely, the *Bacillota*-to-*Bacteroidota* ratio was markedly elevated in the CSFP patient group, reaching 6.56, compared to 4.4 observed in the healthy control group. The reason behind the increase in this ratio is the decreasing amount of *Bacteroidota* in the control group, because *Bacillota* populations were almost same for each group indicated in Figure 3.

At the genus level, 44 genera were significantly different, *p* < 0.05; all were included in Supplementary Figure S1. Notably, the genera Flavobacterium, Gracilibacillus, Novosphingobium, and Weissella exhibited significantly higher abundance in the control group compared to the patient cohort, *p* < 0.01. Moreover, *Gemmiger* was the only significant genus that was more abundant in the patient group compared to the control group.

**Figure 4:**
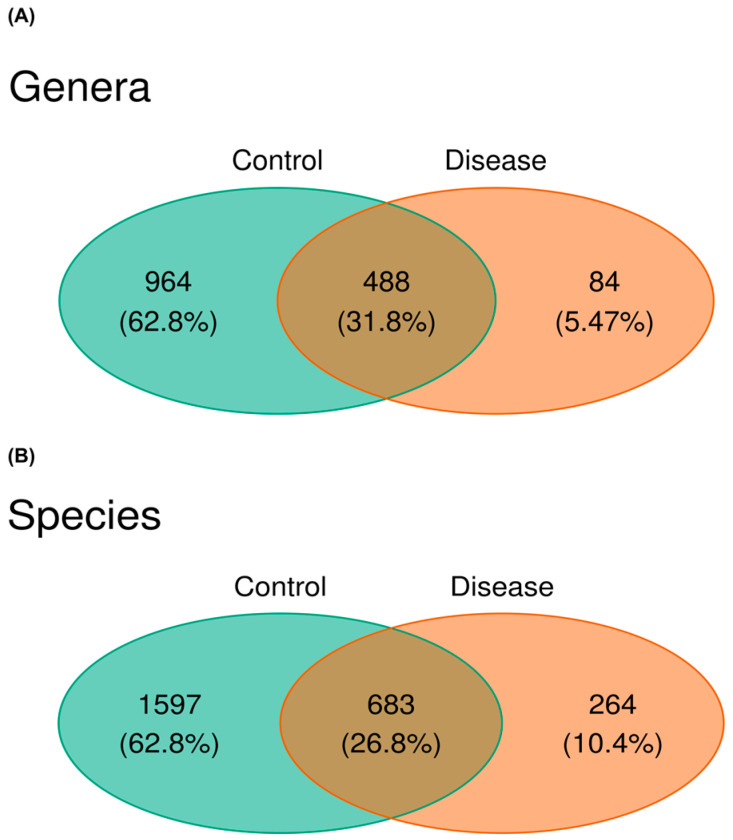
(**A**) The Venn diagram depicts the distribution of OTUs across the genus level and species level. The green section represents control samples, while the red section corresponds to patient samples. (**B**) This visualization illustrates the percentile of both group-specific OTUs and those shared between the groups, providing a detailed overview of the dataset’s composition at the species level.

As a result of the KEGG pathway analysis, the *Gemmiger* genus, which belongs to the *Bacillota* phylum, was found to play a role in selenocompound metabolism. Microbial diversity undergoes dynamic alterations during the onset of a disease, progressively diminishing as the disease advances within the host organism. In our study, results revealed a significant decrease in the gut microbiome diversity of CSFP patients at the genus level, *p* < 0.05; illustrated in Figure 4A. In addition to that, the genera *Roseburia* and *Megasphaera* were identified as key contributors to propionate production. The relative abundance of these genera showed a slight reduction in the disease group, suggesting a potential link to disease-associated metabolic alterations, *p* > 0.05. Only 84 distinct OTUs were observed in diseased individuals, whereas 964 distinct genera were observed in the healthy group. The common OTUs that were observed in both groups were 488. More than half of the significant genera (*Absiella*, *Anaerobutyricum*, *Bacillus*, *Defluviitalea*, *Desulfofundulus*, *Faecalimonas*, *Gracilibacillus*, *Hespellia*, *Kurthia*, *Lachno-bacterium*, *Lachnotalea*, *Lacrimispora*, *Maledivibacter*, *Paralkalibacillus*, *Pediococcus*, *Solibacillus*, *Sporosalibacterium*, *Syntrophomonas*, *Thermoanaerobacterium*, *Thermoclostridium*, *Traorella*, *Virgibacillus*, *Weissella*, and *Xylanibacillus*, *p* < 0.05) belongs to *Bacillota* phylum and together with *Pseudomonadota*, they dominate the genus level of control group with high diversity. Notably, we observed in detail a decrease in microbial diversity at the species level. While 1597 species were only discovered in the control group, 264 species were only present in disease one, as demonstrated in Figure 4B.

Further investigation of species level analysis revealed 25 bacterial species with significant differences in abundance, with the control group exhibiting higher levels than the patient group for all but *Anaerobutyricum*. It was demonstrated in Supplementary Figure S2, *p* < 0.05. *Fournierella massiliensis* and *Gracilibacillus* were the most significant species differing in each group and it is found to be more abundant in controls, *p* < 0.01. Subsequent analysis into pathways associated with the *Anaerobutyricum* genus highlighted its pivotal role in butyrate synthesis, a metabolite essential for human defense mechanisms against various diseases, including cardiovascular conditions [17].

### Association of Trimethylamine on CSFP Disease via Gut Microbiota

**Figure 5:**
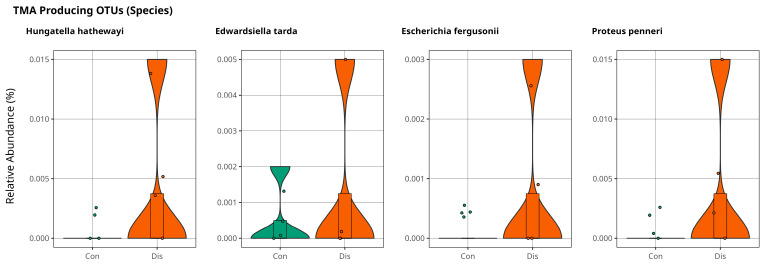
Violin plots illustrate the relative abundance of bacterial species directly involved in TMA production for comparison of control and patient groups, with no statistically significant differences observed (*p* > 0.05).

We conducted an in-depth analysis of metabolite markers implicated in the pathogenesis of cardiovascular disease, identifying elevated levels of trimethylamine N-oxide (TMAO) as a key contributor to disease progression [18]. TMAO is derived from trimethylamine (TMA), a metabolite generated by gut microbiota and subsequently oxidized in the liver. Notably, TMAO is predominantly associated with individuals consuming excessive quantities of red meat, establishing a strong correlation with the development of cardiovascular disorders [19]. *Anaerococcus Hydrogenalis*, *Clostridium Asparaggiforme, Clostridium Hathewayi*, *Clostridium sporogenes*, *Edwardsiella tarda*, *Escherichia Fergusonii*, *Proteus Penneri*, and *Providencia Rettgeri* have been reported as bacterial species found in the gut that contribute to the formation of TMA [20,21]. Our results support the findings of a relatively high abundance of *Hungatella Hathaway*, *Edwardsiella tarda*, *Escherichia Fergusonii*, and *Proteus penneri* in patients with CSFP disease, as illustrated in Figure 5, with *p* > 0.05.

**Figure 6:**
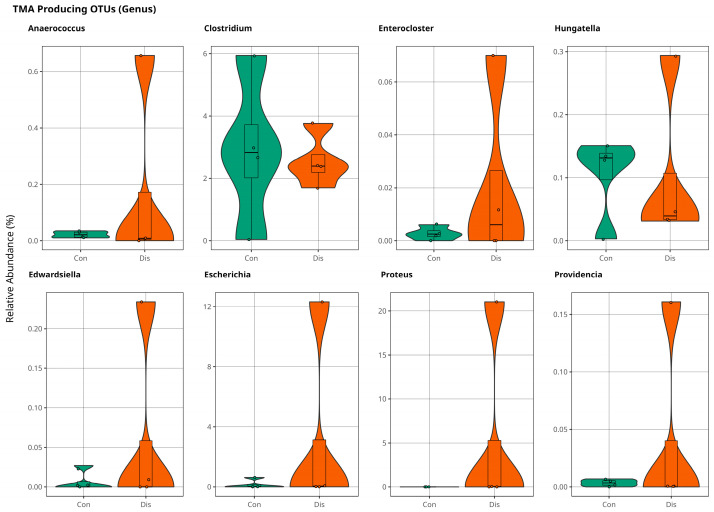
The violin plot illustrates population variations in TMA-producing bacteria between control and patient samples at the genus level, with no statistically significant difference (*p* > 0.05).

At the genus level, *Anaerococcus, Enterocloster, Hungatella, Edwardsiella, Escherichia, Proteus,* and *Providencia* were slightly more abundant in the patient samples indicated in Figure 6. Although these results are not significant, they are promising for further investigations of the coronary slow flow phenomenon to explain disease mechanism of occurrence.

## 4. Discussion

The gut microbiome has a strong effect on the progression of many cardiovascular diseases, such as heart failure, atherosclerosis, hypertension, myocardial fibrosis, and coronary artery diseases, by altering metabolism pathways, especially the trimethylamine N-oxide, short chain fatty acid, and primary and secondary bile acid pathways [22,23]. Elucidating the microbiome and pathway interactions is very important for understanding disease occurrence and its mechanism of action. Studies focusing on this interaction aim to provide a better and early diagnosis and improve more specific treatment approaches. Many studies have been published to explain the microbiome and cardiovascular disease relationship [6,7,8,9,21,24,25]. However, the association between the microbiome and coronary slow flow phenomenon remains unclear. In our study, we analyzed the microbiome differences between patients with coronary slow flow phenomenon and healthy individuals and discovered potential pathways related to disease progression.

We analyzed the microbiome at each level and concluded with results that confirmed some findings from the literature. At the phylum level, we identified 8 significant phyla: *Spirochaetota, Acidobacteriota, Campylobacterota, Elusimicrobiota, Balneolota, Thermotogota, Cyanobacteriota*, and *Myxococcota*. These findings were all more apparent in the control groups than in the patients with coronary slow flow phenomenon. *Bacillota* and *Bacteroidota* accounted for more than 90% of the microbiota in both the healthy and patient groups, but there was no significant difference between the groups. However, the ratio of *Bacillota* to *Bacteroidota* was greater in patients than in healthy subjects. It is caused by the slight decrease in *Bacteroidota*. However, the relative abundance of *Bacillota* is almost the same for two groups. This ratio is elevated in patients with coronary artery disease and is closely linked to various metabolites that play a significant role in the progression of cardiovascular diseases. These metabolites include phosphatidylcholine with a diacyl residue, phosphatidylcholine with an acyl-alkyl, sphingomyelin with an acyl residue, hematocrit, hemoglobin, cholesterol, and LDL cholesterol [21]. A study conducted by Liu Z. and colleagues reported an increase in the ratio of *Bacillota* to *Bacteroidota* in patients with atherosclerosis in one of their studies [26]. Given that CSFP is a form of cardiovascular dysfunction, the observed elevation in the *Bacillota*/*Bacteroidota* ratio among patients relative to healthy controls emerges as a potentially meaningful finding. Notably, several members of the *Bacillota* phylum are known contributors to the production of trimethylamine (TMA), a precursor to TMAO, which has been mechanistically linked to cardiovascular pathogenesis. This correlation strengthens the plausibility that an elevated *Bacillota*/*Bacteroidota* ratio could serve as a non-invasive indicator of CSFP risk when assessed through fecal microbiome profiling. Our findings, which align with the existing literature on cardiovascular diseases, underscore the importance of this microbial ratio as a candidate biomarker and warrant its consideration in future investigations focused on the microbial contributions to CSFP progression.

At the genus level, microbial diversity was significantly lower in the CSFP patient group than in the healthy group. A common microbial output is that the richness of the bacterial population decreases sharply in cardiovascular diseases [27,28,29]. Diversity mostly occurs because of the reduction in beneficial bacterial populations so that pathogens occupy the gut. This leads to the generation of diseases because of the metabolites and proteins secreted by pathogens [30].

*Gemmiger* was found to be the most informative genus for explaining the microbiome and coronary slow flow phenomenon disease, among the 44 statistically significant genera. *Gemmiger*, which belongs to the phylum *Bacillota*, is positively associated with selenocompound metabolism [31,32]. Selenium deficiency has been implicated in a range of pathological conditions including cancer, thyroid dysfunction, inflammatory bowel disease, and cardiovascular disorders primarily through its impact on selenoproteins such as glutathione peroxidases and thioredoxin reductases, which are critical in mitigating oxidative stress and preserving endothelial function. Notably, dietary selenium availability has been shown to correlate strongly with the abundance of *Bacillota* in the gut microbiota. Insufficient selenium intake may disrupt this relationship, leading to compromised vascular repair mechanisms and increased susceptibility to cardiovascular disease [33,34]. Our findings increase the strength of the suggestion that *Gemmiger*, which belongs to *Bacillota*, is a very consequential genus that causes coronary slow flow disease through selenocompound metabolism. Another study revealed that an increasing abundance of *Gemmiger* genera is positively associated with rheumatoid heart disease [33]. All these findings and observations of the high abundance of *Gemmiger* found in patients with CSFP in our study indicate the important role of this genus in cardiovascular disease occurrence. Selenium is an essential chemical for human health, and inadequate amounts of selenium may cause coronary artery diseases [35]. Furthermore, a decrease in propionate-producing bacteria should not be missed out, because increasing the level of the acetate-to-propionate ratio is considered a high-risk factor for cardiovascular disorders [36]. In our case, the relative abundance of *Roseburia* and *Megasphaera* diminished in disease group.

Another significant finding was the high prevalence of *Anaerobutyricum* in patients. It is a butyrate-producing bacteria that enables metabolic regulation and has anti-inflammatory, antioxidant, and anti-obesity effects [37,38]. These short chain fatty acids play important roles in cardiovascular diseases by improving cardiac function and maintaining cardiovascular hemostasis. Butyrate is a four-carbon short chain fatty acid that has anti-inflammatory, antioxidant, anti-obesity, and metabolic regulatory effects. The enrichment of butyrate-producing bacteria regulates the human immune system [39]. Both *Gemmiger* and *Anaerobutyricum* were found to be enriched in the patient group, suggesting a potential compensatory role of the gut microbiota in mitigating endothelial dysfunction through enhanced butyrate production. Butyrate, a short chain fatty acid with well-established anti-inflammatory and vasoprotective properties, has been shown to alleviate endothelial dysfunction in atherosclerosis by downregulating NOX2 expression and reducing reactive oxygen species (ROS) production via the PPARδ/miR-181b signaling axis [40,41,42]. The enrichment of these butyrate-producing genera in CSFP patients may reflect a microbial attempt to restore vascular homeostasis, highlighting the gut microbiota’s potential involvement in counteracting pathophysiological processes linked to coronary microvascular impairment. The involvement of *Gemmiger* in selenium metabolism, along with the butyrate-producing capacities of both *Gemmiger* and *Anaerobutyricum*, may represent key microbial targets for elucidating the pathophysiological mechanisms underlying CSFP. Our genus level findings suggest that these taxa could play a pivotal role in the progression of CSFP, potentially influencing endothelial function through metabolic and immunomodulatory pathways.

On the other hand, there is only one species of butyrate-producing bacteria because the diversity of the microbial community in patients’ guts significantly decreased. Butyrate-producing bacteria belongs to the phylum of *Bacillota* and it is discovered that more than half of the significant genera belongs to *Bacillota* phyla in control groups. This finding indicates the importance of *Bacillota* phylum and the diversity of microbial communities in healthy individuals. Given the reduced microbial diversity observed in CSFP patients, an equally plausible explanation for the enrichment of certain taxa is that it reflects a dysbiotic shift rather than a compensatory microbial adaptation. Accordingly, we acknowledge that our findings are associative in nature, and functional interpretations should be made with caution. To clarify the mechanistic relevance of these microbial patterns, future studies integrating metabolomic profiling, host immune and inflammatory markers, and endothelial function assays will be essential. While our genus level analysis highlights potentially significant microbial signatures, particularly involving Gemmiger and Anaerobutyricum, these results should be viewed as preliminary and hypothesis generating. Functional validation is necessary to determine whether these taxa play a causal role in the pathophysiology of coronary slow flow phenomenon and broader cardiovascular disease processes.

Trimethylamine N-oxide (TMAO) is one of the most extensively studied metabolites implicated in cardiovascular disease (CVD) pathogenesis. Elevated circulating TMAO levels have been directly associated with increased cardiovascular risk. TMAO is formed through a two-step process: dietary precursors such as L-carnitine, betaine, and choline are metabolized by gut microbiota into trimethylamine (TMA), which is subsequently oxidized in the liver to form TMAO [23,43,44]. Several gut microbial taxa are known to contribute directly to TMA production, including the genera *Anaerococcus*, *Clostridium*, *Enterocloster*, *Hungatella*, *Edwardsiella*, *Escherichia*, *Proteus*, and *Providencia*, as well as specific species such as *Anaerococcus hydrogenalis*, *Clostridium asparagiforme*, *Clostridium hathewayi*, *Edwardsiella tarda*, *Escherichia fergusonii*, *Proteus penneri*, and *Providencia rettgeri* [20,21,22].

In our study, we observed a trend toward higher abundance of several TMA-producing genera and species in CSFP patients relative to healthy controls. Notably, *Hungatella hathewayi*, *Edwardsiella tarda*, *Escherichia fergusonii*, and *Proteus penneri* were more prevalent in the CSFP group. Although these differences did not reach statistical significance likely due to the limited sample size, they are consistent with the existing CVD literature and provide important signals worth investigating further. Rather than serving as definitive evidence, these findings should be viewed as preliminary indicators that may guide future mechanistic studies.

To better establish the biological relevance of these microbial shifts in CSFP, future research should incorporate quantification of serum TMAO, correlation of microbial abundances with clinical markers of disease severity, and longitudinal designs to assess causality. Functional assays that explore the metabolic capacity of these bacteria in vitro or in vivo may also help clarify their role in modulating host cardiometabolic pathways. Thus, while our results are currently limited by statistical power, they nonetheless offer a promising foundation for future hypothesis-driven research into the gut microbiota–TMAO–CSFP axis.

Outstanding inferences were made even though we had an insufficient number of samples for analysis to reach a more specific role of the gut microbiome in occurrence of CSFP. CSFP is generally neglected while diagnosing as the clinicians generally check the patients for more common cardiac disorders. Another reason is the exclusion criteria of antibiotic usage; unfortunately, we had to exclude 6 patients from the study after reaching the information of drug usage history of patients. However, the results of this preliminary study were still worthwhile. In particular, the contribution of *Gemmiger* at the genus level to the selenium metabolism pathway makes it a potential microbial biomarker for coronary slow flow phenomenon disease because selenium deficiency is highly correlated with cardiovascular diseases [45,46,47]. We also found that TMA is one of the most important metabolites associated with many cardiovascular diseases [48,49,50,51,52]. In our study, we successfully found the group of gut bacteria that play a role in TMA production during CSFP. Further studies with an increased number of samples are needed to clarify the microbial biomarkers and to understand the pathways that specifically explain their effects on the coronary slow flow phenomenon.

## 5. Conclusions

The development of next-generation sequencing increased the number of studies that remarkably indicate an association with microbiomes. CSFP is a significant cardiovascular disease generally observed in acute coronary syndrome. Although many researchers studied this phenomenon, its association with the microbiome has remained unclear. In this study, we tried to distinguish the gut microbiome of healthy individuals from patients with CSFP. The *Bacilotta*/*Bacteroidota* ratio was higher in patients with CSFP at the phylum level. The *Gemmiger* genus was discovered as the most significant difference between patients and healthy individuals. TMA is one of the most important metabolites associated with cardiovascular diseases. In our study, bacteria species (*Anaerococcus hydrogenalis*, *Clostridium asparaggiforme*, *Clostridium hathewayi*, *Clostridium sporogenes*, *Edwardsiella tarda*, *Escherichia fergusonii*, *Proteus penneri*, and *Providencia rettgeri*) that have a crucial role in TMA production were found to be higher in abundance in patients with CSFP. All these findings are quite promising for further studies to unravel the exact metabolite pathway of the CSFP disease.

## Data Availability

The original contributions presented in this study are included in the article/Supplementary Materials. Further inquiries can be directed to the corresponding author(s).

## Supplementary Materials

The following supporting information can be downloaded at: https://www.mdpi.com/article/10.3390/metabo15070475/s1, Figure S1: Significantly Differentiated Genera; Figure S2: Significantly Differentiated Species

## Abbreviations

The following abbreviations are used in this manuscript:

CSFP	Coronary Slow Flow Phenomenon
TMA	Trimethylamine
SD	Standard Deviation
TSS	Total Sum Scaling
TMAO	Trimethyl-N- Oxide
ROS	Reactive Oxygen Species
