# Supplementary material for: Preliminary Study to Understand the Role of Gut Microbiota in Coronary Slow Flow Phenomenon (CSFP)
Source: Metabolites. 2025 Jul 14;15(7):475. doi: 10.3390/metabo15070475 (PMC12298151; doi:10.3390/metabo15070475)

SUPPLEMENTAL TABLES AND GRAPHS

Supplemental Figure S1. The figure highlights all genera exhibiting significant differences between healthy controls (Con) and patients with CSFP disease (Dis) ($p < 0.05$).

Supplemental FİgureS2. The figure demonstrates the relative abundance of statistically significant bacterial species of healthy controls (Con) and patients with CSFP disease (Dis) ($p < 0.05$).

Supplemental Figure S1. The figure highlights all genera exhibiting significant differences between healthy controls (Con) and patients with CSFP disease (Dis) ($p < 0.05$).

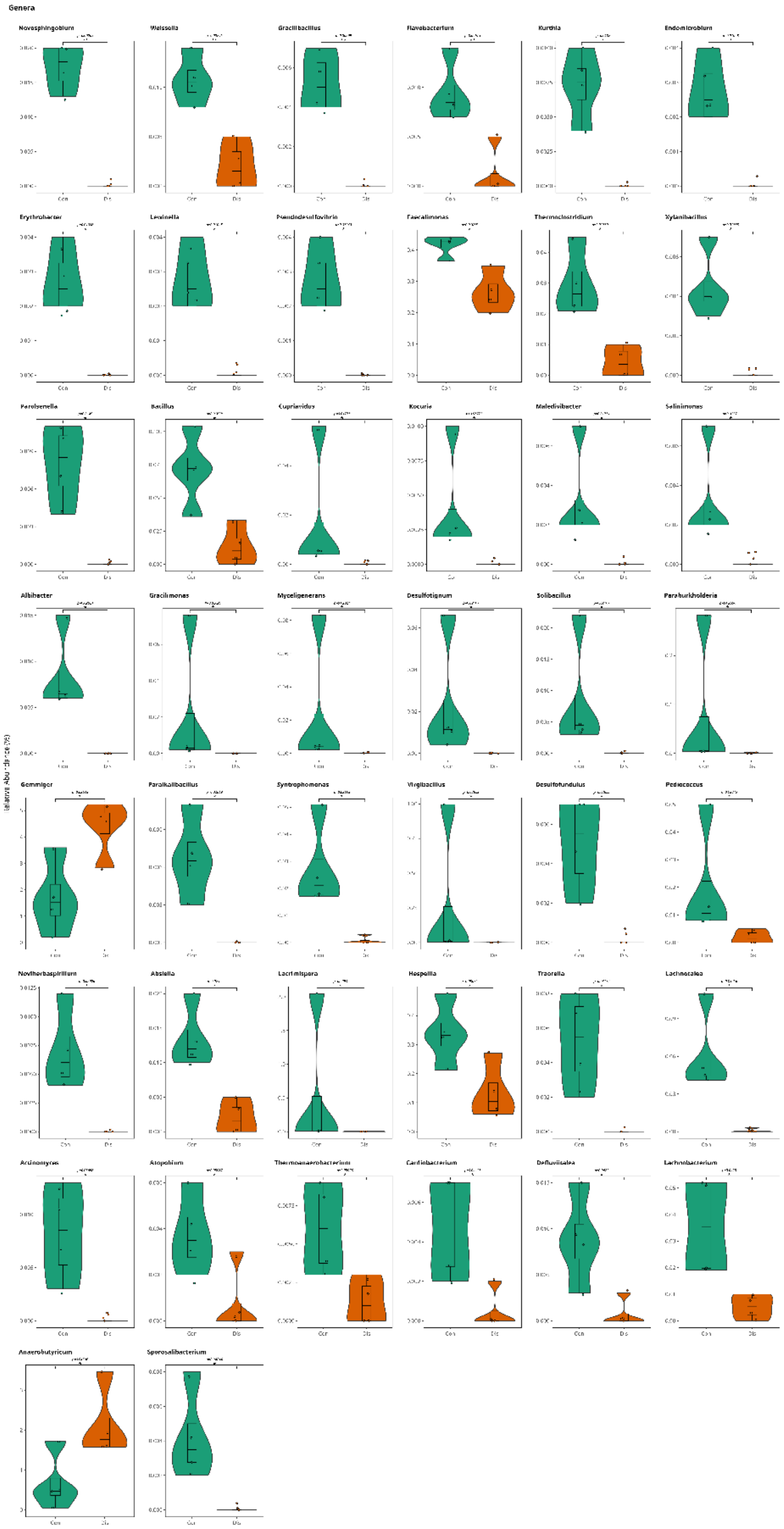

Supplemental Figure S2. The figure demonstrates the relative abundance of statistically significant bacterial species of healthy controls (Con) and patients with CSFP disease (Dis) ($p < 0.05$).

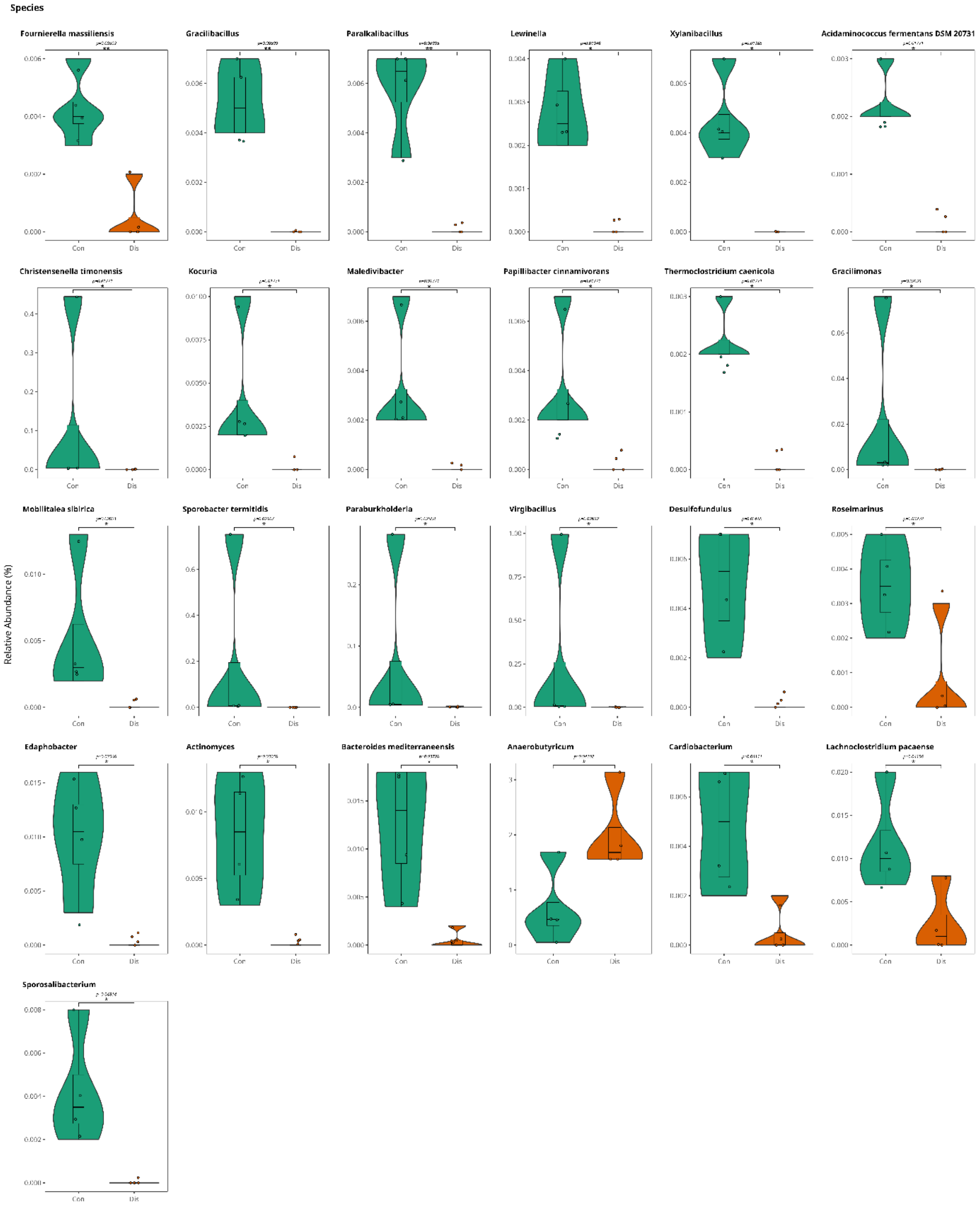

Supplement: Supplementary file 1 [file metabolites-15-00475-s001.zip › Supllementary File1.pdf]
